# Stranguary as a Sign of Pregnancy

**Published:** 1860-08

**Authors:** B. McC

**Affiliations:** Dubuque, Iowa


### STRANGUARY AS A SIGN OF PREGNANCY.

By B. McC——, M. D., of Dubuque, Iowa.

Any facts which can make more certain medical diagnosis, may be considered worthy the notice and attention of medical men.

It is with this view that I have made the following notes of two cases which came under my observation.

April 28th, 1857, I was called to see a young woman who had been married upon the 6th of the same month. She was about seventeen years of age. I had been called on account of most severe stranguary with which she had been troubled for several days.

But as she had from infancy been troubled with a difficulty of retaining urine a proper length of time, she thought it only a little different phase of the same old trouble, until it became so severe that there was constant pain and desire to urinate without the ability of discharging more than a few drops at a time, and that with great pain and distress.

I prescribed decoctions of Uva-Ursi and of Buchu without relief, and finally put her upon camphor, which gave more relief than anything which was tried, though warm fomentations were repeatedly applied over the region of the bladder. The difficulty continued to recur from time to time for some two weeks, when it finally disappeared.

I could not readily see any cause for the difficulty, as the patient seemed to be an unusually healthy woman, and by the inspection of the urine I could detect no evidences of urinary deposits of any kind.

The thought of pregnancy being the cause suggested itself to my mind; I examined several authors in reference to that point, and found no one mentioning stranguary or dysuria as an early sign of conception; and besides, upon inquiry, I learned that she had enjoyed the connubial bed but two nights. But the after history of the case proved pregnancy to have been the cause.

She was married April 6th, had had her menses about one week previous to marriage, and did not menstruate afterwards, and was delivered of a strong female child on the 26th of the following December, being thirty-seven weeks and five days from the marriage.

Again, August 4th, 1858, I was called to see a young married lady, who complained of a constant desire to urinate, but could only get rid of a few drops, as she expressed it, and that with very great pain, more or less tenderness, etc., accompanying the discharge.

She had been married some two or three months, had been *regular*, and had menstruated only ten days before I saw her.

As I could find no good cause for this sudden attack, in an otherwise healthy young woman, and one who had never had any difficulty in the urinary or generative organs; and remembering the case above related, I ventured to express to the husband my opinion that *possibly* it might be that conception had taken place, and that it was the cause of the present difficulty.

The diagnosis thus expressed proved correct, and she was delivered of a healthy female infant, May 1st, A. D., 1859, being just forty weeks from the menstruation which occurred ten days previous to my first attendance.

I have, since these two cases, continued to look at the authors to which I have had access, and have found stranguary or dysuria mentioned but by one as an evidence of pregnancy in its early stage, and that is by Dr. Blundell, as given in his testimony in the Gardiner Peerage Case, (Beck's Med. Jurisprudence, vol. 1, p. 593,) where he says:

"I saw a case a few days after impregnation; there were symptoms of irritation about the bladder and adjacent parts, and the catamenia were absent." He had no doubt but these symptoms arose from impregnation.

It will be noticed in the report of my cases that there had not been sufficient time from the last menstruation for the recurrence of the menstrual function, and yet the history of both cases proved that the diagnosis of conception would have been safe and correct.

The question arises, was this a merely accidental coincidence, which might lead astray in the next case? Or, is strangaury really a phenomenon, which may be taken under certain circumstances as a sign of impregnation before the first month is passed?

Any facts from the profession on this subject would be interesting to your correspondent.

